# A Stochastically Optimized Two-Echelon Supply Chain Model: An Entropy Approach for Operational Risk Assessment

**DOI:** 10.3390/e25091245

**Published:** 2023-08-22

**Authors:** Konstantinos Petridis, Prasanta Kumar Dey, Amit K. Chattopadhyay, Paraskevi Boufounou, Kanellos Toudas, Chrisovalantis Malesios

**Affiliations:** 1 Department of Business Administration, University of Macedonia, 54006 Thessaloniki, Greece; k.petridis@uom.edu.gr; 2 Aston Business School, Aston University, Birmingham B4 7ET, UK; p.k.dey@aston.ac.uk; 3 Department of Applied Mathematics & Data Science, College of Engineering and Physical Sciences, Aston University, Aston Triangle, Birmingham B4 7ET, UK; 4 Department of Economics, National and Kapodistrian University of Athens, 10559 Athens, Greece; 5 Department of Agribusiness and Supply Chain Management, Agricultural University of Athens, 75, 11855 Athens, Greece; kstoudas@aua.gr; 6 Department of Agricultural Economics and Rural Development, Agricultural University of Athens, 75, 11855 Athens, Greece; malesios@aua.gr

**Keywords:** green supply chain management, supply chain risk model, stochastic models, noise

## Abstract

Minimizing a company’s operational risk by optimizing the performance of the manufacturing and distribution supply chain is a complex task that involves multiple elements, each with their own supply line constraints. Traditional approaches to optimization often assume determinism as the underlying principle. However, this paper, adopting an entropy approach, emphasizes the significance of subjective and objective uncertainty in achieving optimized decisions by incorporating stochastic fluctuations into the supply chain structure. Stochasticity, representing randomness, quantifies the level of uncertainty or risk involved. In this study, we focus on a processing production plant as a model for a chain of operations and supply chain actions. We consider the stochastically varying production and transportation costs from the site to the plant, as well as from the plant to the customer base. Through stochastic optimization, we demonstrate that the plant producer can benefit from improved financial outcomes by setting higher sale prices while simultaneously lowering optimized production costs. This can be accomplished by selectively choosing producers whose production cost probability density function follows a Pareto distribution. Notably, a lower Pareto exponent yields better supply chain cost optimization predictions. Alternatively, a Gaussian stochastic fluctuation may be proposed as a more suitable choice when trading off optimization and simplicity. Although this may result in slightly less optimal performance, it offers advantages in terms of ease of implementation and computational efficiency.

## 1. Introduction

Operational risk is the risk of loss because of ineffective or failed internal processes, people, systems, or external events, which can disrupt the flow of business operations. An inviolable aspect of a business organization is the distribution of supply lines, both on the input side of the business as well as in relation to its output deliverables, together with the supply chain management (SCM) of its overall throughput. Due to rapid economic globalization, the majority of operations, ranging from manufacturing to transportation sectors and from warehousing to the customer base, are conducted by supply chain contractors or third-party logistics (3PL) companies. Recent research in logistics developments predicts that, in the foreseeable future, approximately 80% of economic transactions will be based on services. Thus, the better the design of supply chain operations, the better the service level the customers will experience. Currently, for the majority of products transported and sold throughout the world, customers rarely subscribe to brand loyalty; thus, any stock clearance may result in a reduction in sales and future loss of income for firms [1,2,3].

Furthermore, entering the 21st century, the business environment is becoming more and more challenging because of the worldwide effort to meet the SDGs’ challenges [4]. Green supply chain management (GSCM) integrates environmental thinking into supply chain management, creating a sustainable supply chain [5]. As noted in the relevant literature (see, e.g., [6,7,8,9,10,11,12,13,14]) companies are under immense pressure to adopt GSCM practices that are driven towards the environment by a combination of external factors (government rules and legislation; environmental concerns and regulation; social and environmental responsibility; customer awareness, pressure, and support; supplier pressure and willingness; global climate pressure) and internal factors (green image; global marketing; competitiveness; economic beliefs or cost reduction benefits; investor and shareholder pressure; employee motivation; health and safety issues; waste management issues) towards meeting SDGs, as they directly affect customer choice [15]. Furthermore, many studies are currently focusing on discussing the implementation of GSCM in different sectors of the economy and/or specific countries (see, e.g., [16,17,18,19,20,21,22,23,24,25,26]).

While value chain management has hugely benefitted from paradigmatic studies in the realm of supply chain theories, involving deterministic variation of associated variables and parameters (see, e.g., [27,28,29,30,31,32,33]), very little has been done in connection with the impact of stochastic perturbations in probabilistically predicting the qualitative and quantitative assessment from the supply chain model (see, e.g., [34,35]). For a comprehensive and up-to-date review on the subject we refer the interested reader to [36] Optimizing the design of a supply chain provides an “ideal” image of the real situation that, by construction, is not amenable to conventional mathematical modeling [29,37]. The issue here is the randomized nature of the data produced from the supply chain performance profile, which are mathematically categorized as stochastic in design. Although stochastic programming models have been proposed where each unique scenario is associated with a corresponding probability of occurrence, such models have not essentially incorporated the full range of stochastic effects, e.g., market uncertainty, decision making uncertainty, etc., in their formulation, which is undertaken in our model. Real-world situations entail mismatches in the operations conducted among the nodes of the supply chain, in a way that affects the levels of upstream and downstream decisions [38].

Identifying stochasticity in the operations is not enough, though, to provide real and stochastic decisions [39,40]. The majority of the works proposed in the supply chain literature incorporate modeling uncertainty as a token of sensitivity analysis involving changes in the parameters of the models, e.g., through case scenarios (bounds, demand, supply, etc.) (see e.g., [35,41,42]). However, such approaches do not adequately represent the misalignments of the conducted operations in the supply chain. For instance, [41] proposed to address uncertainty in production demand though a scenario planning approach wherein parameters varied with changing scenarios, whereas [40] extended previous attempts by proposing algorithms that could handle a larger number of alternative scenarios for the model’s parameters. Other approaches (e.g., [43]) included assigning a single distribution to demand uncertainty, followed by a scenario-based analysis. See also [44,45] for similar approaches. However, the scenario-based approaches inherently cannot identify all potential outcomes of uncertainty parameters of interest, as their structure is not inherently defined through a probabilistic Bayesian approach, much like a stochastic adaptation of the same could be. The information mismatch is another phenomenon that is not addressed adequately within the frameworks of the aforementioned types of stochastic models. Such inadequate information routing is expected to have a knock-on effect on the supply chain network design due to the stochastic nature of the information flow pattern. This work adopting an entropy approach addresses this key knowledge gap, focusing on the variable nature of the stochastic fluctuations involved and how it could affect the probabilistic prediction from this type of (stochastic) supply chain kernel.

In this work, the problem of designing an optimal supply chain network design is tackled by incorporating different types of noise into the variables of the study. Hence, the present work sets the foundation for a new optimization routine in which the noise representations are taken from a variety of well-known statistical distributions. Our proposed model extends previous works on multi-echelon supply chain design, using [46] mixed-integer non-linear programming (MINLP) by introducing stochastic fluctuations in the supply chain structure to account for the degree of demand uncertainty more effectively. Our proposed method adds an additional stochastic model to [46] single deterministic MINLP model, hence extending the former to a sequential deterministic-stochastic model.

Cost optimization, based on a cost function, is then performed by optimizing with respect to the stochastic variables. We should note that some of the parameters themselves could be stochastic, in addition to the variables. Thus, the primary objective of this paper is to develop a mathematical model of a supply chain that accounts for all of the inherent stochastic fluctuations of the system and its parameters.

This will be under the auspices of a two-state modular structure that has the special feature of stochastic noise being embedded into the design of the supply chain network. Assuming different types of noise representations in terms of their respective probability density functions (specifically, we assume the Gaussian, Lognormal, and Pareto distributions), the supply chain model is analyzed to quantify which of these PDFs ensure cost minimization through an optimization rationale perpetrated across the entire supply chain network. Fluctuations are not formulated through different parameter distribution representations but are directly introduced through the variables. Model two represents a stochastic ensemble of a generalized sampling procedure, in the spirit of Sample Average Approximation [47,48,49]. This drives target-specific research questions, such as what the nature of the distribution function conforming to the “ideal” situation is, and which distribution function could precipitate an increasing cost.

The rest of the paper is structured as follows. Section 2 outlines prior literature on modeling of the supply chain network and the optimal supply chain network design problem. Section 3 explains the proposed methodology. This is followed by the presentation of findings (Section 4). Section 5 discusses the range of empirical findings in line with earlier studies and concludes with a few suggestions.

## 2. Literature Review

The optimal supply chain network design (SCND) problem has been extensively examined in the literature. The majority of the proposed models are drawn from mathematical programming disciplines and are roughly divided into two categories: (a) steady state models and (b) multi-period models [50].

More specifically, [51] proposed a nonlinear programming (NLP) model providing an integrating framework for the facility location and inventory allocation problem with cost discounts. A two-phase approximation approach was deployed as a solution to provide numerical results that could demonstrate the impact of different simulated data to the supply chain decisions and cost. [33] propose a multi-echelon supply chain model that includes suppliers, plants, and distribution centers and aims at minimizing the total cost of the supply chain. The proposed methodology involves sensitivity analysis to show that the customer demand parameter has the greatest impact on the optimal solution. [31] propose a deterministic model for the supply chain uncertainty in the demand. The suggested model assumes that returned items from the customers can be remanufactured at a fixed rate.

Choi et al. [27] study the supply chain scheduling and co-ordination problem comprising multiple suppliers, a single warehouse operator, a single manufacturer, and multiple retailers. Fattahi et al. [30] investigate the supply chain network design and planning for a multi-commodity and multi-layer network over a planning horizon with multiple periods, in which the demands of customer zones are considered price dependent through the development of a mixed-integer linear programming (MILP) model.

Similarly, in another development directed towards fluctuation incorporation, ref. [41] used a mixed-integer linear programming model wherein both binary and continuous variables are considered with the objective of assigning uncertainty in the structure of the hierarchical variables, e.g., demand as deterministic uncertainty in their respective numbers, without explicit incorporation of statistical stochastic terms. The first are used for network representation, while the latter for facility capacity and flows of goods throughout the channels of the supply chain network [32]. Similar models have been proposed, considering the demand uncertainty and measuring the customers’ service level through the calculation of lead time and normally distributed demand (see, e.g., [46,52]). The formulation of an agile or flexible supply chain network with the use of a heuristic algorithm as a solution procedure has been also proposed by [53] as a means of incorporating certain non-deterministic fluctuations perpetrating changed functionalities in the supply chain.

Closed-loop supply chains (CLSC) are generally used to model the reusability and recycling of products (ICT, food, etc.) In a more recent work, ref. [54] proposed a fuzzy MILP model to capture the uncertainty in demand, cost, and other parameters. Similar modeling approaches have been proposed in the literature using mathematical programming techniques for the optimal closed-loop supply chain network design (CLSCND) [55,56].

Recent works focus mostly on biomass-based supply chain networks due to a global turn towards bioenergy production. In their work, ref. [57] proposed a data envelopment analysis (DEA) based algorithm for optimal biomass supply chain network design. An optimal design of a forest supply chain network has been proposed by [58]. In this work, the authors employed a Lagrangian relaxation algorithm [59] to design the fuel–wood supply chain, considering demand uncertainty. The optimal design of a biofuel supply chain network has been also examined using a Monte Carlo simulation approach to provide a sensitivity analysis for various parameters [35].

In another setting, the use of multiple objective functions may be seen as providing a more realistic approach to real-world problems. In such domains, multi-objective programming (MOP) models have been traditionally employed, including the optimal design of chemical supply chains [60], biofuel/biomass supply chains [28,61], in forest supply chains [62], and considering green supply chains with environmental factors [63].

The introduction of noise realization has been examined in many production–allocation systems (including the supply chain network design problem). The main modeling method for noise representation is optimal control. In these lines, [64] have proposed a multi-echelon control model to describe a production–allocation supply chain network. In their work, the authors assumed that noise corrupted demand and system delays. A popular approach is based on the value of stochastic solution (VSS) [65] to compare relative contributions between deterministic and stochastic amplitudes within the remit of the same model. Our sequential model, too, is inspired by this VSS approach.

A game theoretical model is proposed by [66], where through a collaborative approach, a noise (read fluctuation) reduction scheme was propounded. Noise, in terms of uncertainty, has also been modeled through different demand and supply scenarios identifying disruptions to the production process [67]. A decision support system is proposed by [68], where the performance of service level or customer satisfaction was examined through a simulation study. Uncertainty has been modeled by adding noise to the demand parameter or by sampling from statistical distributions.

One recent article titled “Stochastic Inventory Control in a Multi-Echelon Supply Chain: A Review” [69] examines the existing literature on stochastic inventory control in multi-echelon supply chains. It delves into various mathematical models, optimization techniques, and decision-making approaches employed to manage uncertainty in inventory levels across different stages of the supply chain. The review emphasizes the need for robust inventory policies and coordination mechanisms to mitigate the impact of stochasticity.

Johnson et al. [70] provide a comprehensive analysis of supply chain risk management, encompassing stochastic events. It discusses the identification, assessment, and mitigation of risks associated with stochasticity, such as demand volatility, supplier disruptions, and natural disasters. The article emphasizes the importance of building resilient supply chains through effective risk management strategies.

Liu et al. [71] focus on managing disruptions caused by stochastic events in supply chains. They explore strategies such as redundancy, flexibility, and collaboration that can help to mitigate the impact of disruptions and improve supply chain robustness. The article also discusses the role of technology, such as real-time monitoring and predictive analytics, in enhancing supply chain resilience.

In the realm of supply chain management, various studies have been conducted to explore different aspects and challenges. Ref. [72] conducted a study to analyze the impact of financial risk on the manufacturer–supplier relationship in a two-echelon supply chain. They developed a multi-objective decision model for supplier selection and order allocation, aiming to maximize the manufacturer’s total profit while minimizing the financial risk faced by selected suppliers. The study considered foreign exchange risk, default risk, market risk, and price fluctuation risk, and explored three case scenarios to understand the behavior of suppliers in response to different financial risks, both in the short and long term.

Building on the concept of risk aversion in supply chains, ref. [73] examine a two-echelon supply chain with two competing manufacturers and one retailer. One manufacturer adopted sustainable technology to reduce carbon emissions under cap-and-trade regulations, while the other followed traditional business practices. The study considered two configurations involving risk-neutral and risk-averse agents and analyzed operational decisions using a retailer–leader game optimization approach under the mean variance framework. The results showed that risk-averse agents benefited from low-scale risk aversion, and low carbon emissions were attainable when the underlying manufacturer had small or moderate risk aversion.

In a different approach, ref. [74] explore the application of thermodynamics in describing the behavior of economic and financial systems. They discuss the first and second laws of thermodynamics and construct a mathematical model for a constant price process. The focus is on examining the dynamics of economic processes using thermodynamics principles. However, more specific findings and conclusions from the research were not provided in the summary.

Additionally, ref. [75] conduct a literature review on risk and disruption management in production–inventory and supply chain systems. They reviewed works that considered real-life risk factors, such as imperfect production processes, disruptions in production, supply, demand, and transportation. The review emphasized the mathematical models and solution approaches used to address these problems, both in hypothetical and real-world scenarios. The review concluded by discussing future research directions in this area.

Furthermore, ref. [76] proposed a nonlinear programming (NLP) model, providing an integrating framework for the facility location and inventory allocation problems with cost discounts. They deployed a two-phase approximation approach as a solution to provide numerical results that demonstrate the impact of different simulated data on supply chain decisions and cost.

More realistic, explicit incorporations of multiplicative noise routines have rarely come across in the relevant literature. This is partly due to computational difficulty, and to the minimalistic nature of most problems considered.

## 3. Methods

### 3.1. Model Concepts

This section outlines our entropy modeling approach. We consider two models: deterministic and stochastic. In the first case, the model is solved in the deterministic way without any additional noise in the variables, while in the second stochastic approach, different uncertainty representations are modeled with varying noise distributions (i.e., normal, lognormal, and Pareto). In the following, the general supply chain network framework hypothesized for the application of our modeling is presented, followed by a detailed illustration of the deterministic and stochastic supply chain models.

#### 3.1.1. Supply Chain Network Framework

**Figure 1:**
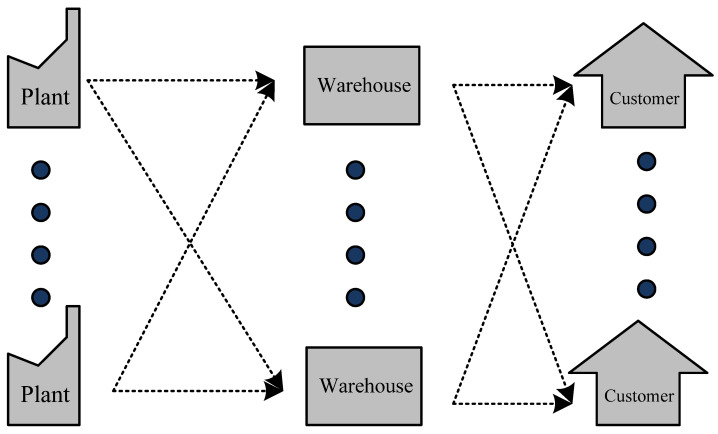
Multi-stage, multi-echelon supply chain network.

In this paper, a multi-stage multi-echelon model is presented that hierarchically incorporates functional interactions between plants, warehouses, customer zones, and thereby multiple echelons in turn. The initial and final links of the supply chain are considered fixed and only the quantities of products produced (for plants) and products transported (for customers) are provided. A graphical representation of the hypothesized supply chain network is provided in Figure 1 below.

In the framework outlined above (Figure 1), the warehouses are assumed uninstalled. Thus, based on the intermediate link, the supply chain network is constructed. The mathematical structure is a sequential solution involving two models. In the first model, the optimal design of the supply chain network is calculated based on stochastic demand, assuming that it is is normally distributed [46]. This first model generates decision outputs that serve as inputs for the second model that we now describe. Using decision inputs from the first model, the second model computes possible shortfalls in demand. The expected lead time is then computed to estimate quantities providing knowledge on stock out situations.

**Figure 2:**
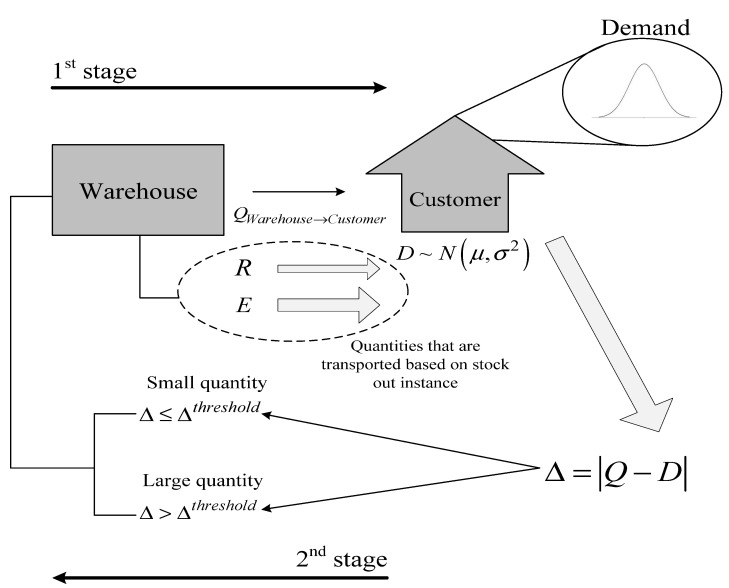
The two-stage supply chain model.

The stock out instances, which are defined as the absolute difference between demand and the quantities of products delivered to customer, are divided into two categories based on a threshold decided by the decision maker (DM). In many cases, a stock out instance may not just affect the service level, and therefore the perception of the customers towards a specific product but may lead to penalty costs due to a contract clause (this is especially applicable in the food supply chain industry). In addition to holding inventory, since warehouses are assumed to serve as production facilities as well, the inventory can serve as raw materials to cover the deficits in demand. The magnitude of production quantities is assigned to a corresponding production cost that is added to the total cost function of the 1st stage. Due to stock out instances, the expected lead time (*ELD*) keeps increasing. The fact that warehouses are used as production facilities in the supply chain reduces the expected lead time but may increase the overall cost significantly, leading to a trade-off between cost and service quality. The aforementioned procedure is graphically represented in Figure 2 below.

#### 3.1.2. Deterministic Model

The optimization model presented here provides levels of decisions for the quantities of a single product, even though extensions can also be considered. The model presented here is an extension of the mixed-integer nonlinear programming (MINLP) model proposed by [46]. The Petridis model is a deterministic structure resembling our model one. As mentioned, the output of model one serves as the input of model two, thereby making it a sequential deterministic → stochastic model that allows for a more realistic and generalized market perspective.

For the mathematical representation of the optimization model, each node (stage) is assigned an index. The first stage (plants) is denoted by *i*, the second (warehouses) by *j,* and the third (customer zone) by *k*. In the following context, the constraints of the problem are presented. As each plant has a limited capability (i.e., given its resources, raw material, etc.), the production capability of each plant *i* is upper and lower bounded as given below.

(1) $$P_{i}\leq P_{i}^{U},\forall i$$

(2) $$P_{i}\geq P_{i}^{L},\forall i$$

In Equations (1) and (2), $P_{i}^{U}$ and $P_{i}^{L}$ define the upper and lower bounds of production, which are assumed to be known a priori (see Appendix A for a detailed description of all models’ variables and parameters along with abbreviations).

Next, as the produced quantities are transported downstream (from the production to the end customer), the following mass balance constraint is considered, modeling the fact that the produced quantities by plant *i* should equal to the quantities transported to warehouse *j*:

(3) $$P_{i}=\underset{j}{\sum }Q_{ij},\forall i$$

Moreover, the quantities entering the warehouse node should be equal to those that exit that node (from warehouse *j* to customer zone *k*):

(4) $$\underset{j}{\sum }Q_{ij}+I_{j\;}=\underset{k}{\sum }Q_{jk},\forall k$$

Finally, the quantities transported throughout the supply chain end to the customers’ end should be greater than or equal to the demand of each customer. The demand (*D_k_*) is assumed to follow a statistical distribution that is already known.

(5) $$\underset{k}{\sum }Q_{jk}\geq D_{k},\forall k$$

As mentioned previously, the warehouse facilities are not known a priori and are decided after setting up the optimization model. Generally, decisions of “yes” or “no” type are introduced with binary variables. The connection between the plant *i* and warehouse *j*, as well as between customer *k* and warehouse *j*, is assumed to exist only if warehouse *j* exists.

(6) $$X_{ij}\leq Y_{j},\forall i,j$$

(7) $$X_{jk}\leq Y_{j},\forall j,k$$

In (6) and (7) constraints, the binary variables $Y_{j}$ are used for modeling whether warehouse *j* will be installed in position *j* or not and $X_{ij}$ to model the connection between nodes *i* and *j*.

The quantities (*Q*) are transported from one node to another only if the corresponding connection exists.

(8) $$Q_{ij}\leq Q_{ij}^{U}\cdot X_{ij},\forall i,j$$

(9) $$Q_{jk}\leq Q_{jk}^{U}\cdot X_{jk},\forall j,k$$

Finally, the warehouse quantities (*W*) can be computed through the following constraints (a Lagrange multiplier type approach):

(10) $$W_{j}\geq a_{j}\cdot \underset{i}{\sum }\left(Q_{ij}+I_{j}\right),\forall j$$

(11) $$W_{j}\leq W_{j}^{U}\cdot Y_{j},\forall j$$

We seek to minimize the overall cost, through the following objective function (*TC*):

(12) $$TC=\underset{i}{\sum }c_{i}^{P}\cdot P_{i}+\underset{i}{\sum }\underset{j}{\sum }c_{ij}^{VTR}\cdot Q_{ij}+\underset{i}{\sum }\underset{j}{\sum }c_{ij}^{FTR}\cdot X_{ij}+\underset{j}{\sum }\underset{k}{\sum }c_{jk}^{VTR}\cdot Q_{jk}\;+\underset{j}{\sum }\underset{k}{\sum }c_{jk}^{FTR}\cdot X_{jk}+\underset{j}{\sum }c_{j}^{IN}\cdot Y_{j}$$

In the objective function (12), the first term represents the production cost, the second and fourth terms represent variable transportation costs, while the third and fifth terms account for the fixed variable costs, and the final term represents the installation (or capital) cost.

As there may be shortfalls in demand (unsatisfied demand) due to misinformation or scheduling, natural disasters that may disrupt this chain, etc., the following parameter is introduced:

(13) $$\Delta_{k}=\left|D_{k}-\underset{j}{\sum }Q_{jk}^{*}\right|,\forall k$$

Parameter $\Delta_{k}$ serves as a critical threshold to define the level of unforeseen expenses to be expected in emergent conditions. This threshold is divided into the following two ranges:

$\left.\left[\Delta^{L},\Delta^{M}\right.\right]$ and $\left(\Delta^{M},\Delta^{U}\right]$; the threshold is divided into a low valued range of supply insufficiency as indicated by the first range, and in a high values of supply insufficiency, corresponding to very large shortfalls in providing the demanded quantity. The examined ranges are defined as: $\Delta^{L}=min_{k}\left\{\Delta_{k}\right\}$, $\Delta^{U}=max_{k}\left\{\Delta_{k}\right\}$ while $\Delta^{M}=\frac{\Delta^{L}+\Delta^{U}}{2}$.

In Equation (13), the absolute difference between the demand representation of each customer *k* and the transported quantities computed from the previous stage (stage 1) is shown. In order to model the magnitude of the failure in customer satisfaction, the following constraints are additionally introduced:

(14) $$\Delta^{L}\cdot \lambda_{k}\leq Q_{k}^{U}\leq \Delta^{M}\cdot \lambda_{k},\forall k\;$$

(15) $$\Delta^{Μ}\cdot \zeta_{k}\leq Q_{k}^{O}\leq \Delta^{M}\cdot \zeta_{k},\forall k$$

(16) $$\lambda_{k}+\zeta_{k}=1,\forall k$$

Constraints (14) and (15) are introduced to model the deficit in demand under or over a pre-specified threshold. Binary variables $\lambda_{k}$ and $\zeta_{k}$ are mutually exclusive, as any shortfall in demand can be characterized as over or under a specific threshold but cannot fall in both categories, as indicated in (16).

Finally, in stock out instances, several corrective actions should be undertaken to improve the service level without significantly increasing the cost. After the introduction of these corrections, the new objective function is as follows (see Appendix B for a detailed description of the associated corrections to the original objective function):

(17) $$TC1=TC+\underset{j}{\sum }\underset{k}{\sum }c_{jk}^{PO}\cdot E_{jk}+\underset{j}{\sum }\underset{k}{\sum }c_{jk}^{PU}\cdot R_{jk}+\underset{k}{\sum }\sigma \cdot \sqrt{ELD_{k}}$$

where the Expected Lead Time (ELD) is computed based on the following equality:

(18) $$ELD_{k}=T^{u}\cdot P_{k}\left(Q^{O}\right)\cdot \zeta_{k}+T^{l}\cdot P_{k}\left(Q^{U}\right)\cdot \lambda_{k}\;$$

In the objective function presented in (17), *σ* is the standard deviation of unsatisfied demand for customer *k* such that: $\sigma =\frac{\sqrt{\sum_{k}{\left(\Delta_{\kappa }-{\bar{\Delta }}_{k}\right)}^{2}}}{n-1}$ and ${\bar{\Delta }}_{k}$ is the mean unsatisfied demand.

From the above analysis, the following levels of decision are derived from each stage:

- 1st stage:
  ○ Produced and transported quantities;
  ○ Selected warehouses and capacity;
  ○ Supply chain network;
  ○ Demand deficit.
- 2nd stage:
  ○ Stock out and overstocking probabilities;
  ○ Expected lead time (*ELD*);
  ○ Quantities that should be produced to cover unsatisfied demand.

#### 3.1.3. Stochastic Model

To introduce inherent uncertainty into the model, we draw from the well-established fluid mechanics theories [76,77], wherein flows along the lines of symmetry (e.g., on the axis of a cylinder) are known to be perfectly deterministic while the ones closer to the edges, e.g., boundary layer Taylor-Couette flows, show statistical fluctuations due to boundary layer stress. For such systems, the complete solutions of the model are taken as the linear superposition of the deterministic flow together with a stochastic noise term, where the noise distribution will characterize the actual system concerned. From the perspective of our two-tiered model structure, model 1 will serve the role of the deterministic (MINLP) solution that then will be noise mixed to define model 2. The solution of the entire system is then the sum of the MINLP deterministic solution together with an additive stochastic noise, as detailed below:

(19) $$\tilde{P_{i}}=P_{i}+\eta ,\;\forall i\;$$

(20) $$\tilde{Q_{ij}}=Q_{ij}+\eta ,\;\forall i,j$$

(21) $$\tilde{Q_{jk}}=Q_{jk}+\eta ,\;\forall j,k$$

(22) $$\begin{array}{c} for\;e=1,\ldots n\\ \;for\;e^{\prime }=1,\ldots ,n\\ \;\;{\tilde{X}}_{ee^{\prime }\;}=X_{ee^{\prime }}+\eta_{ee^{\prime }}\\ \;end\\ end \end{array}$$

Equations (19)–(21) represent a De Dominicis-Martin [77] representation, where the deterministic variables have been replaced by their stochastic equivalents (*η*). As discussed before, the total solution is then a linear sum of the deterministic component (MINLP) together with a noise term (*η*). This replacement procedure is mathematically described in (22).

The optimization kernel was executed over multiple time loops within a Matlab-based architecture.

### 3.2. Implementation of Deterministic and Stochastic Models

**Figure 3:**
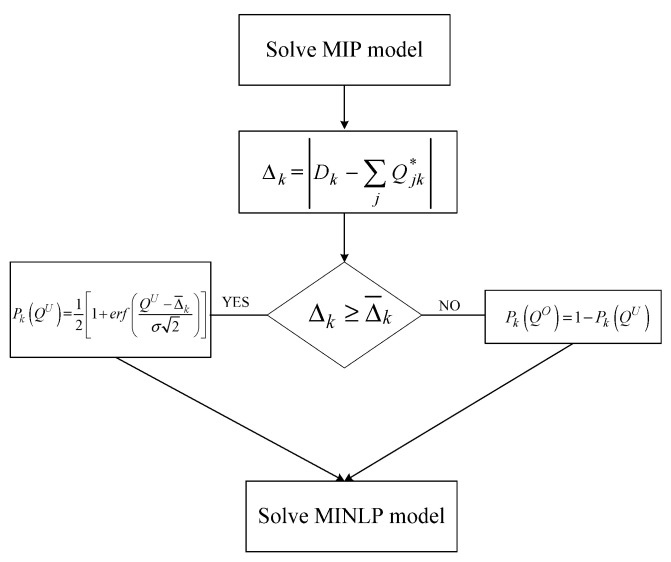
Flowchart of the deterministic implementation.

**Figure 4:**
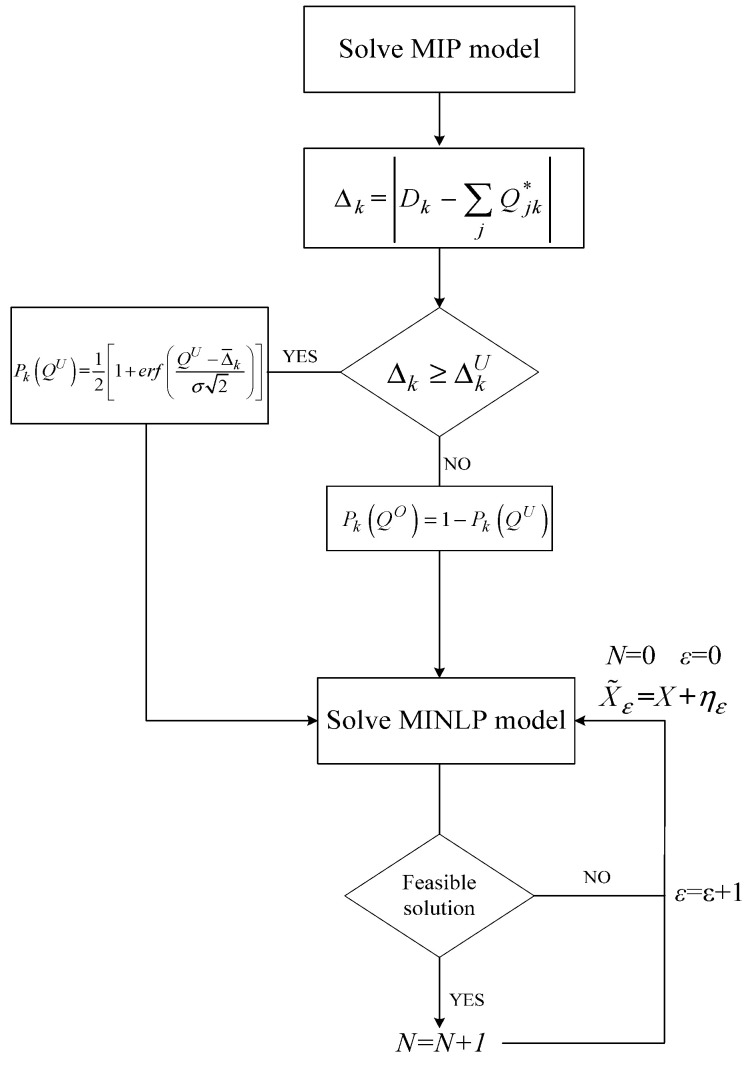
Flowchart of the stochastic implementation.

In the current section, a graphical representation of the implementation steps of the deterministic and stochastic models is presented in Figure 3 and Figure 4, respectively. In the first case, the model is solved deterministically. As seen in Figure 3, initially the mixed-integer programming (MIP) problem is solved, while the shortfalls in the demand are computed for each customer. To measure the magnitude of stock out instances, it is assumed that if $\Delta_{\kappa }$ is more than the average stock out quantities, there is a large deficit in meeting demand and thus the expected lead time for demand satisfaction will be larger than in the case in which this deficit is of less magnitude. The final step, as seen in Figure 3 below, is the calculation of the MINLP model, wherein the expected lead time, the probabilities of over- and understocking instances, and the levels of variables are provided.

On the contrary, in the stochastic case proposed and developed in the current study, the MINLP model is solved for different noise representations for the basic variables that concern the production and transportation of flows as described in Equations (19)–(21). The introduction of noise into the variables is implemented using procedure (22). For each new variable, the MINLP may yield a feasible solution (optimal, local optimal of integer) or an infeasible solution. Unfeasible solutions might represent a significant loss for the supply chain. This needs to be carefully and independently addressed in a separate work. A counter is introduced to model each choice where the MINLP model yields a feasible solution, as presented in Figure 4 below.

#### Risk Assessment

**Figure 5:**
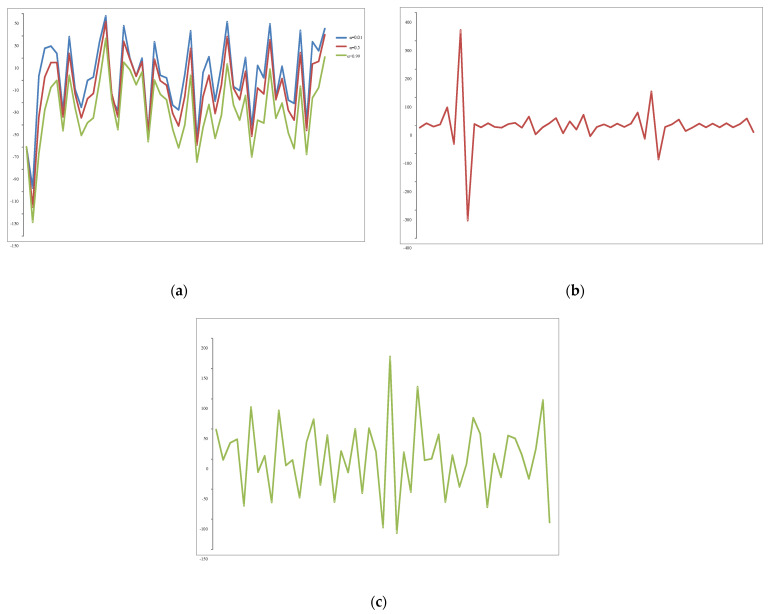
Noise representations: (**a**) Pareto noise for *α* = 0.01 (blue line), *α* = 0.5 (red line), *α* = 0.99 (green line); (**b**) lognormal noise; (**c**) Gaussian noise.

In this sub-section, the different noise functions utilized to model the stochastic part of our proposed supply chain network model are presented. The representations of different distributions of noise used are presented and graphically illustrated in Figure 5 below:

- Gaussian noise;
- Lognormal noise;
- Pareto noise for various alpha levels (*α* = 0.01; *α* = 0.5; *α* = 0.99).

## 4. Results

**Figure 6:**
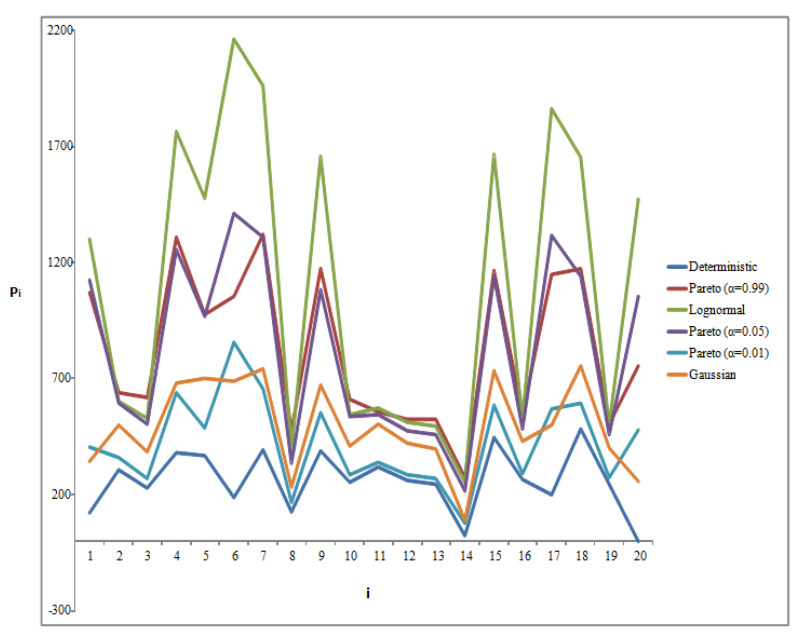
Results for *P_i_* under the different noise representations and in comparison to the deterministic model.

In the current section, we focus on the comparative results between the supply chain models including noise (stochastic model) and the typical model without noise (deterministic model). The quantities $Q_{ij}$ and $Q_{jk}$ denote the transported quantities from plant *i* to warehouse *j* and from warehouse *j* to customer *k*, respectively. $P_{i}$ is the production capacity for plant *i*. We want to see which stochastic model is closest to the deterministic fixed-point model. To provide the most suitable representation of differences between estimated quantities for the various models, and due to the reasonably large number of nodes considered at each echelon, heatmap plots are presented. In our estimation, we have assumed this nodal number to be 20 $\left|I\right|=\left|J\right|=\left|K\right|=20$. In Figure 6 below, the results of $P_{i}$ estimations for the different noise realizations are shown. Our stochastic modeling shows that the optimized cost under the assumption of a Pareto noise distribution (for Pareto exponent a→0) comes closest to the deterministic prediction, while Pareto distributions with larger exponent values as well as the other distributions (i.e., lognormal and Gaussian) lead to poor cost optimization schemes.

**Figure 7:**
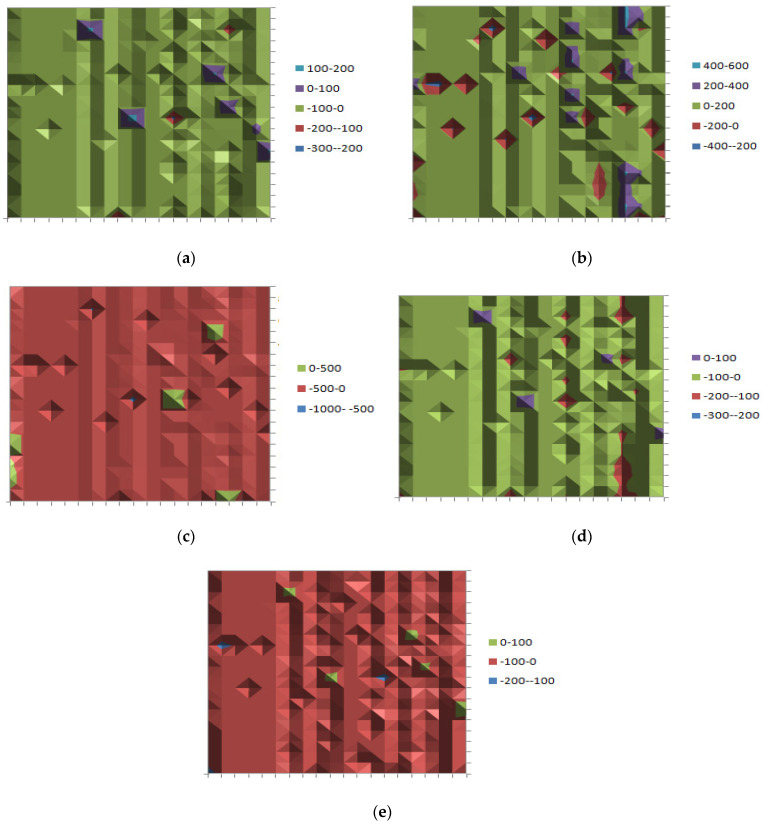
Heatmaps for the differences of deterministic values of *Q_ij_* − Q*_ij_ for (**a**) Pareto noise with *a* = 0.01, (**b**) Pareto noise with *a* = 0.5, (**c**) Pareto noise for *a* = 0.99, (**d**) Gaussian noise, (**e**) lognormal noise.

Next, in Figure 7 below, the results comprising of the differences between the deterministic variable ($Q_{ij}^{det}$) and stochastic ones ($Q_{ij}^{noise}$), which correspond to the results of transported quantities from plant *i* to warehouse *j*, are presented. If these values are closer to 0, then it can be concluded that the addition of the specific noise does not have a significant impact on the overall supply chain network design. In Figure 7a, in presence of Pareto noise with *a* = 0.01, most of the area lies in the range of [−100, 100] (green and purple color). This means that the fluctuations from the deterministic values of variable $Q_{ij}$ can range from −100 to 100. Figure 7b demonstrates that the fluctuations are increasing to the range [−400, 200] when considering Pareto noise with *a* = 0.5. Figure 7c depicts the differences between a deterministic model and stochastic model with Pareto noise (*a* = 0.99). Although most of the area lies in the range of [−500, 500], the “bumps” reduce when compared to the Pareto (*a* = 0.5). Figure 7d (Gaussian case) clearly shows fluctuations, most of which lie in the range of [−100, 100]. Finally, in Figure 7e there is approximately the same image as in Figure 7d, but nevertheless most of the area lies in the range of [−100, 0].

To provide an overall measure of the comparison results presented above, the standard deviation (*σ*) of the differences derived after the introduction of each noise representation with the deterministic ones is presented in the following Table 1.

Best performance, based on the overall measure of *σ*, is shown by the Pareto (*a* = 0.01) model (*σ* = 27.92), followed closely by the Gaussian stochastic specification (*σ* = 29.36). The worst fit, on the other hand, is shown for the lognormal noise stochastic model (*σ* = 98.97). Note that depending on the value of the Pareto exponent, the distribution will have a sharp or a long-tailed decay. On the other hand, a Gaussian system is finitely correlated and also symmetric, which is easier to analyze but the cost is accuracy.

## 5. Discussion and Conclusions

Effective supply chain management is a key concern for companies, especially in the realm of the environmental concerns of green supply chain management promoting Agenda 2030. The optimal design of a supply chain network may be oriented from the customer’s perspective (namely “pull” systems) or from production’s perspective (namely “push” systems) [78]. In “pull” systems, demand drives production while, in the second case, production is fixed based on demand estimation. Such deductions are likely to be modified once the time evolution of a supply chain is considered [79]. However, in most cases the optimal design of the supply chain network is constructed around parameter values that approximate the upper and lower bounds of the transported quantities. In several studies, stochasticity has been introduced either as different scenarios or by integrating a statistical distribution into the parameter (expected value), to capture the characteristic of uncertainty [40].

None of these approaches, though, reflect the absolute real situation as the uncertainty is measured based on the parameter and not based on the variable space, aside from the fact that such subroutines can only lead to implicit uncertainty measures at best and inaccurate predictions at worst. The previous statement can be easily understood with the following example. If the well-known ‘bullwhip’ effect occurs [80], then the variable that corresponds to the quantities that are transported from the final node of the supply chain to the customer’s site has to report this malfunction in the supply chain operation. Assuming further that stochastic fluctuations are driving the demand line, the production is adjusted based on the new value of demand; however, the information mismatch is not taken into account. The integration of noise into the variables instead of the parameters resets the problem at its fundamental base and remodels the information mismatch.

The probabilistic nature of this study on generic non-equilibrium systems, where the inputs are essentially stochastic, can only be assessed on a statistical manifold. The principle of maximum entropy stipulates that an equilibrium system that is largely unperturbed by ambient perturbations is characterized by a state of maximum entropy. That is the underlying thematic of this study, which is to arrive at stable equilibrium fixed points from a study of dynamically evolving non-equilibrium processes. This has been done using a two-echelon model, which is the most minimalist description conceivable. Such a state of maximized entropy incorporates all inherent stochastic fluctuations, inclusive of (business and risk) uncertainties that a real business model needs to accommodate. To achieve this, we have considered a two-stage supply chain modeling approach where, in the first stage, a MILP model is solved to provide the solutions for the second model; the levels of solutions that are derived from the first model concern the construction of the supply network and solutions that correspond to quantities transported throughout the supply chain. In the second model, the expected lead time is measured based on the amount of unsatisfied demand (Δ). Imposing thresholds on “small” or “large” Δ, the network is reconstructed providing additional information regarding the capacity of the facilities and the magnitude of products that need to be constructed, as it is assumed that warehouses serve as small production plants to minimize the expected lead time and therefore increase service level. Specifically, we propose a mechanism involving stochastically varying production and transportation costs in the supply chain network. In doing this, three types of stochastic noise are examined: normal (Gaussian), lognormal, and Pareto. The obtained results indicated large differences between the fitted models, with the lognormal noise model producing a larger fluctuation from the actual situation (deterministic), while a smaller fluctuation is observed for the Pareto noise, and especially the one with the smallest exponent (*α* = 0.01), followed by the Gaussian model.

Generally, we see that a very low Pareto noise (*α* ≈ 0.01) is very well risk managed, since this is very close to a deterministic model. Moreover, note that as a general stochastic disturbance, a Gaussian fluctuation shows *σ* very close to that of Pareto for *α* = 0.01. This establishes that most real models will on average be risk averse if the stochasticity follows a normal distribution. Another interesting finding from the results is that Pareto models have a large fluctuation depending on the choice of *α* parameter. From a managerial perspective, this structure offers numerous advantages. First, this clearly tells us that real uncertainty measures are likely to be non-Gaussian rather than Gaussian, which probabilistically optimizes the risk percentage. Second, very low Pareto exponents effectively enumerate a correlation-independent uncertainty. In other words, a lower Pareto exponent points to the limit of uniform distribution, whereas a large Pareto exponent will point to an improbable event. Since the target of this analysis is to identify the impact of factors that dominate the degree of departure of a supply chain network from an idealized Gaussian model, the Pareto exponent value works as a statistical descriptor providing this information. This means we now have an enumerator that clearly separates the risk associated with one measure from that of the other. For example, uncertainty in worker numbers due to civil or political unrest is known to affect the product line delivery rate. Using our model, we will now be able to establish by how much. Finally, a practical manager needs to rank uncertainties in order of their merit. Progressively lower values of the Pareto exponent offer a statistical measure of ranking.

The derived cost function showcases how to incorporate such stochasticity in a supply chain model and what eventual benefits one may derive out of it. As a tailored example, we show that a producer may benefit from a better return only through a suitable selective choice of producers whose production cost probability density function has a Pareto distribution. Such a study can have a significant impact on any overall supply chain cost due to the linearly increasing objective function. While such stochastic optimization is not unknown in the realm of statistical mechanics (Spall, 2003), the mapping is an altogether new concept in supply chain literature; an approach that has the prospect of coming up with rich dividends in the future.

## Figures and Tables

**Table 1:** Standard deviation (*σ*) of the differences between the deterministic value of variables and noise representation.

$\mathrm{Noise}\;\mathrm{Representation}\;Q_{ij}^{det}-Q_{ij}^{noise}$	Standard Deviation (*σ*)
Pareto Noise (*a* = 0.01)	27.92
Pareto Noise (*a* = 0.5)	70.16
Pareto Noise (*a* = 0.99)	97.65
Gaussian Noise	29.39
Lognormal Noise	98.97

## Appendix A. Index and Variable Explanation

**Index**	
i	Plant
j	Warehouse
k	Customer
**Continuous Variables**	
TC	Total supply chain cost
$P_{i}$	Produced quantities in plant i
$Q_{ij}$	Transported quantities from plant i to warehouse j
$Q_{jk}$	Transported quantities from warehouse j to customer k
$E_{jk}$	Transported quantities from warehouse j to customer k that exceed a certain level (high)
${\tilde{P}}_{i}$	Produced quantities in plant i with noise representation
${\tilde{Q}}_{ij}$	Transported quantities from plant i warehouse j with noise representation
${\tilde{Q}}_{jk}$	Transported quantities from warehouse j to customer k with noise representation
$R_{jk}$	Transported quantities from warehouse j to customer k that exceed a certain level (low)
$W_{j}$	Capacity of warehouse j
$ELD_{k}$	Expected lead time of customer k
$P_{k}\left(Q^{O}\right)$	Stock out probability of customer k
$P_{k}\left(Q^{L}\right)$	Overstocking probability of customer k
$\Delta_{k}$	Deficit in demand satisfaction for customer *k*
**Binary Variables**	
${Χ}_{ij}$	1 if the corresponding connection between plant i to warehouse j exists, 0 otherwise
${Χ}_{jk}$	1 if the corresponding connection between warehouse j to customer k exists, 0 otherwise
$Y_{j}$	1 if warehouse j is selected, 0 otherwise
$K_{jk}$	1 if small quantities will be delivered from warehouse *j* to customer *k* due to large demand deficit, 0 otherwise
$\Omega_{jk}$	1 if large quantities will be delivered from warehouse *j* to customer *k* due to large demand deficit, 0 otherwise
$\lambda_{k}$	$1\;\mathrm{if}\;\mathrm{the}\;\mathrm{deficit}\;\mathrm{in}\;\mathrm{demand}\;\mathrm{satisfaction}\;\mathrm{lies}\;\mathrm{in}\;\mathrm{the}\;\mathrm{interval}\;\left[\Delta^{L},\;\Delta^{U}\right]$, 0 otherwise
$\zeta_{k}$	$1\;\mathrm{if}\;\mathrm{the}\;\mathrm{deficit}\;\mathrm{in}\;\mathrm{demand}\;\mathrm{satisfaction}\;\mathrm{lies}\;\mathrm{in}\;\mathrm{the}\;\mathrm{interval}\;\left[\Delta^{U},\;\Delta \right]$, 0 otherwise
**Parameters**	
$P_{i}^{U}$	Upper bounded production of plant *i*
$P_{i}^{L}$	Lower bounded production of plant *i*
$Q_{ij}^{U}$	Maximum capacity of transported quantities from plant *i* to warehouse *j*
$Q_{ij}^{L}$	Minimum capacity of transported quantities from plant *i* to warehouse *j*
$Q_{jk}^{U}$	Maximum capacity of transported quantities from warehouse *j* to customer *k*
$Q_{jk}^{L}$	Minimum capacity of transported quantities from warehouse *j* to customer *k*
$I_{j}$	Inventory held at warehouse *j*
$D_{k}$	Demand of customer *k*
$\Delta_{k}$	Stock out quantity in customer *k*
${Τ}^{u}$	Maximum time for product delivery
${Τ}^{l}$	Minimum time for product delivery
$\alpha_{j}$	Coefficient relating quantity at capacity at warehouse *j*
$\beta_{j\kappa }$	Production rate for quantities stored at warehouse *j* that will be delivered to customer *k* in order to cover the high deficit in demand satisfaction.
$\gamma_{j\kappa }$	Production rate for quantities stored at warehouse *j* that will be delivered to customer *k* in order to cover the low deficit in demand satisfaction.
**Cost Parameters**	
$c_{i}^{P}$	Production cost of plant *i*
$c_{ij}^{VTR}$	Variable transportation cost of plant *i* to warehouse *j*
$c_{ij}^{FTR}$	Fixed transportation cost of plant *i* to warehouse *j*
$c_{jk}^{VTR}$	Variable transportation cost of warehouse *j* to customer *k*
$c_{jk}^{FTR}$	Fixed transportation cost of warehouse *j* to customer *k*
$c_{j}^{IN}$	Installation cost of warehouse *j*
$c_{jk}^{PO}$	Production cost of small quantities that will be manufactured in warehouse *j* and will be delivered to customer *k*
$c_{jk}^{PU}$	Production cost of large quantities that will be manufactured in warehouse *j* and will be delivered to customer *k*

## Appendix B. Implementation of Stock Out Instances in the Deterministic Model

In stock out instances, several corrective actions should be undertaken to improve the service level without significantly increasing the cost. In the case wherein the deficit in demand belongs to an interval above the predetermined threshold, new quantities are produced from the fixed inventory kept in warehouse *j* ($I_{j}$), along with the corresponding deficit of demand for this particular customer *k*.

If the quantity that corresponds to low values of supply insufficiency and binary variable that corresponds to the low range become 1 for some indices of customers of *k* (*k*_1_), then $\lambda_{\kappa_{1}}=1$ and, based on the predetermined range in (14), warehouses that are also assumed to be production plants holding inventory used for manufacturing purposes will have to produce additional quantity equal to *R_jk_* as seen in (A2). Constraint (A2) provides a value that corresponds to the quantity to be produced based on constraint (A1), as binary variable $K_{j\kappa }$ takes a value of 1 if $\lambda_{\kappa }$ equals 1. In that case, the quantity that will eventually be produced by warehouse j in order to facilitate a medium stock out occurring at customer k should be more than $\gamma_{j\kappa }\cdot H_{j\kappa }$; $\gamma_{j\kappa }$ stands for the production coefficient of warehouse j for each customer k, and $H_{j\kappa }$ is a minimum level of inventory stored for the production of the necessary quantity in warehouse j in order to facilitate a medium stock out occurring at customer k Constraint (A3) models the occurrence of a large stock out instance, while the production quantity that is needed to be sent to customer k from warehouse j is defined as $E_{j\kappa }$, and should be more than the warehouse’s j production rate ($\beta_{j\kappa }$) multiplied by the sum of the overall inventory held at warehouse j and stock out occurred in customer k as in (A4).

(A1) $$K_{jk}\leq \lambda_{k},\forall k\;$$

(A2) $$R_{jk}\geq \gamma_{jk}\cdot H_{jk}\cdot K_{jk},\;\forall j,k$$

(A3) $$\Omega_{jk}\leq \zeta_{k},\;\forall j,k$$

(A4) $${Ε}_{jk}\geq \beta_{jk}\cdot \left(I_{j}+\Delta_{k}\right)\cdot \Omega_{jk},\;\forall j,k$$

Constraint (5) is now reformulated as follows:

(A5) $$\underset{j}{\sum }Q_{jk}+Q_{k}^{U}+Q_{k}^{O}=D_{k},\forall k$$

Based on (A5), the probability of stock out or overstock can be computed. If demand is normally distributed $D_{k}\sim N\left(0,\sigma^{2}\right)$, then $\Delta_{k}$ is assumed to be normally distributed as well (as the difference of two random variables that are normally distributed. Thus, stock out and overstocking probabilities are introduced with the following constraints:

(A6) $$P_{k}\left(Q^{U}\right)=\frac{1}{2}\left[1+erf\left(\frac{Q^{U}-\bar{\Delta_{k}}}{\sigma \sqrt{2}}\right)\right],\forall k$$

(A7) $$P_{k}\left(Q^{O}\right)=1-P_{k}\left(Q^{U}\right),\forall k$$

The service level in the supply chain can be easily quantified with the expected lead time, namely, the amount of time needed for a product to be delivered to the customer after order placement.

The expected lead time (ELD) is computed based on the following equality:

(A8) $$ELD_{k}=T^{u}\cdot P_{k}\left(Q^{O}\right)\cdot \zeta_{k}+T^{l}\cdot P_{k}\left(Q^{U}\right)\cdot \lambda_{k}$$

This leads to the new objective function as follows:

(A9) $$TC1=TC+\underset{j}{\sum }\underset{k}{\sum }c_{jk}^{PO}\cdot E_{jk}+\underset{j}{\sum }\underset{k}{\sum }c_{jk}^{PU}\cdot R_{jk}+\underset{k}{\sum }\sigma \cdot \sqrt{ELD_{k}}$$
